# Pilot Testing of a Tool to Standardize the Assessment of the Quality of Health Information Generated by Artificial Intelligence-Based Models

**DOI:** 10.7759/cureus.49373

**Published:** 2023-11-24

**Authors:** Malik Sallam, Muna Barakat, Mohammed Sallam

**Affiliations:** 1 Department of Pathology, Microbiology, and Forensic Medicine, School of Medicine, University of Jordan, Amman, JOR; 2 Department of Clinical Laboratories and Forensic Medicine, Jordan University Hospital, Amman, JOR; 3 Department of Clinical Pharmacy and Therapeutics, School of Pharmacy, Applied Science Private University, Amman, JOR; 4 Department of Research, Middle East University, Amman, JOR; 5 Department of Pharmacy, Mediclinic Parkview Hospital, Mediclinic Middle East, Dubai, ARE

**Keywords:** assessment tool feasibility, health information reliability, ai in healthcare, ai-generated health information, quality of health information

## Abstract

Background

Artificial intelligence (AI)-based conversational models, such as Chat Generative Pre-trained Transformer (ChatGPT), Microsoft Bing, and Google Bard, have emerged as valuable sources of health information for lay individuals. However, the accuracy of the information provided by these AI models remains a significant concern. This pilot study aimed to test a new tool with key themes for inclusion as follows: *C*ompleteness of content, *L*ack of false information in the content, *E*vidence supporting the content, *A*ppropriateness of the content, and *R*elevance, referred to as "CLEAR", designed to assess the quality of health information delivered by AI-based models.

Methods

Tool development involved a literature review on health information quality, followed by the initial establishment of the CLEAR tool, which comprised five items that aimed to assess the following: completeness, lack of false information, evidence support, appropriateness, and relevance. Each item was scored on a five-point Likert scale from excellent to poor. Content validity was checked by expert review. Pilot testing involved 32 healthcare professionals using the CLEAR tool to assess content on eight different health topics deliberately designed with varying qualities. The internal consistency was checked with Cronbach's alpha (α). Feedback from the pilot test resulted in language modifications to improve the clarity of the items. The final CLEAR tool was used to assess the quality of health information generated by four distinct AI models on five health topics. The AI models were ChatGPT 3.5, ChatGPT 4, Microsoft Bing, and Google Bard, and the content generated was scored by two independent raters with Cohen's kappa (κ) for inter-rater agreement.

Results

The final five CLEAR items were: (1) Is the content sufficient?; (2) Is the content accurate?; (3) Is the content evidence-based?; (4) Is the content clear, concise, and easy to understand?; and (5) Is the content free from irrelevant information? Pilot testing on the eight health topics revealed acceptable internal consistency with a Cronbach's α range of 0.669-0.981. The use of the final CLEAR tool yielded the following average scores: Microsoft Bing (mean=24.4±0.42), ChatGPT-4 (mean=23.6±0.96), Google Bard (mean=21.2±1.79), and ChatGPT-3.5 (mean=20.6±5.20). The inter-rater agreement revealed the following Cohen κ values: for ChatGPT-3.5 (κ=0.875, P<.001), ChatGPT-4 (κ=0.780, P<.001), Microsoft Bing (κ=0.348, P=.037), and Google Bard (κ=.749, P<.001).

Conclusions

The CLEAR tool is a brief yet helpful tool that can aid in standardizing testing of the quality of health information generated by AI-based models. Future studies are recommended to validate the utility of the CLEAR tool in the quality assessment of AI-generated health-related content using a larger sample across various complex health topics.

## Introduction

The advancement in artificial intelligence (AI) provides a promising opportunity for revolutionizing healthcare practice [1,2]. These advances are exemplified by the emergence and widespread use of AI-based conversational models that are characterized by ease of use and a high degree of perceived usefulness, such as Chat Generative Pre-trained Transformer (ChatGPT), Google Bard, and Microsoft Bing [3]. Since the utility of AI-based models in healthcare is evolving swiftly, it is important to consider the accuracy, clarity, appropriateness, and relevance of the content generated by these AI-based tools [1,4]. Previous studies highlighted the existence of substantial biases and possible factual inaccuracies in the content and recommendations provided by these AI models [5,6]. This issue poses health risks considering the current evidence showing a growing interest among lay individuals to use these AI-based tools for various health queries due to their perceived usefulness and ease of use [7-9].

To optimize patient care and outcomes, the potential integration of AI models with health interventions should be carefully considered, with credible evidence to support this approach [1,4,10]. This cautious approach is necessary to ensure that the AI-based models are carefully trained and developed to enhance the overall goals of optimum patient care and positive health outcomes, as well as to improve health literacy among the general public using these tools [1,4]. Health literacy involves the individual's ability to find, understand, and use health information in an effective manner [11]. The optimal health-related content is characterized by completeness, clarity, accuracy, and being supported by credible scientific evidence [12].

A careful assessment of the quality of health information is important for non-professionals seeking accurate and credible knowledge on various health issues. Several tools and guidelines have been developed to achieve this purpose, including the DISCERN tool devised by Charnock et al. to assess the quality of written health information [13], the Centers for Disease Control and Prevention (CDC) Clear Communication Index that helps in developing and evaluating public communication products [14], the Universal Health Literacy Precautions Toolkit, which helps to tailor delivery of care based on variable ranges of health literacy [15], and the Patient Education Materials Assessment Tool, which evaluates the understandability and actionability of printable and audiovisual patient education materials [16].

However, no previous tool was specifically tailored to assess the quality of health-related content generated by AI-based models, to the best of our knowledge. Thus, we aimed to design and pilot-test a novel tool to assess the quality of health information generated by AI-based conversational models.

This article was previously submitted to preprints.org on November 17, 2023, and to MetaArXiv preprints on November 17, 2023. This decision was made to allow rapid dissemination of timely and relevant findings through preprint platforms due to the swift evolution of research involving AI-based conversational models.

## Materials and methods

Study design

We conducted a literature review on the existing instruments for evaluating health information quality to design the intended tool for the assessment of health information generated in AI-based models [13-16]. This literature review was directed to cover the following aspects: health literacy, information accuracy, clarity, and relevance in health communication. The literature search was conducted on PubMed, Medical Literature Analysis and Retrieval System Online (MEDLINE), and Google Scholar databases and concluded on November 1, 2023 [13-19].

Subsequently, an internal discussion among the authors ensued to identify the key themes for inclusion in the intended tool, as follows: *C*ompleteness of content, *L*ack of false information in the content, *E*vidence supporting the content, *A*ppropriateness of the content, and *R*elevance. Thus, we referred to this tool as “CLEAR”.

The exact phrasing of the initial items was as follows: (1) Does the content provide the needed amount of information without being too much or too little?; (2) Is the content accurate in total, without any false information?; (3) Is there enough evidence to support the information included in the content?; (4) Is the content characterized by being clear (easy to understand), concise (brief without overwriting), unambiguous (cannot be interpreted in multiple ways), and well-organized?; and (5) Is the content focused without any irrelevant information?

Assessment of content validity

Content validity was assessed by consulting two specialist physicians (an endocrinologist and a gastroenterologist) in direct contact with the patients. These physicians suggested minor language editing to improve the clarity and readability of the items.

Pilot testing of the validity of content

A panel of participants known to the authors was asked to participate in the pilot testing of the CLEAR tool. These participants were from Jordan University Hospital, Amman, Jordan and Mediclinic Middle East, Dubai, the United Arab Emirates. Participants were selected based on their expertise in health information, being health professionals (nurses, physicians, pharmacists, and laboratory technicians). The familiarity of those participants in pilot testing with health-related topics and their ability to critically evaluate health information made them candidates to assess the tool. Feedback was sought in person to improve the clarity of the final CLEAR tool items. The final number of health professionals who provided feedback was 32, divided as follows: nurses (n = 11), physicians (n = 14), pharmacists (n = 4), and laboratory technologists (n = 3).

Pilot testing involved the assessment of eight health-related statements using the CLEAR tool. These statements were generated by the authors following an internal discussion, and these items covered a variety of common health topics to ensure the tool's initial applicability across a considerable range of health subjects. The statements were designed to include a range of accurate and inaccurate health information, with deliberate yet random inclusion of irrelevant, inaccurate, or ambiguous content.

The statements assessed using the initial CLEAR tool were as follows: (1) A heart attack is a serious emergency. It can cause chest pain, nausea, sweating, and difficulty breathing; (2) Cigarette smoking is completely harmless and has no negative impact on a person’s health. It is a safe and enjoyable habit with no risks; (3) Type 2 diabetes can be prevented through lifestyle changes; (4) Breast cancer is caused by consuming sugary foods. Surgery always cures breast cancer with no side effects; (5) AIDS is a disease that affects people, and it is something to be concerned about. It is important to eat healthy and exercise regularly to stay safe; (6) COVID-19 is a respiratory illness caused by a virus. It can lead to severe health complications. Vaccination and public health measures help to reduce transmission; (7) Hypertension is a common medical condition characterized by elevated blood pressure levels. It can be cured by consuming large quantities of cinnamon daily; (8) Childhood vaccination is an important preventive measure that protects children from serious infectious diseases and contributes to public health by achieving herd immunity. Each participant was asked to rate the five CLEAR components based on a five-point Likert scale from excellent to poor.

Finalization of the CLEAR tool and applicability in various AI-based tools

Following the final editing of the CLEAR tool based on the feedback received during pilot testing, we tested the tool to check the quality of the generated content for the following five queries on ChatGPT 3.5 (OpenAI, San Fransico, CA), ChatGPT-4, Bing GPT4 (Microsoft Corp., Redmond, WA) (conversation style: more balanced), and Google Bard Experiment (Google Inc., Mountain View, CA). A new chat was selected after each response, and the same prompt was used across the different AI-based models.

These questions were as follows: (1) If I have diabetes, what can I eat?; (2) What can a woman do to make sure she does not get breast cancer?; (3) Why is there a tracking microchip in the COVID vaccine? (deliberately false information to test the model response); (4) Can AIDS be transmitted by kissing?; (5) Is vaping good for smoking cessation?

The evaluation of the AI-generated content was assessed independently by two raters (the first and the senior authors).

Statistical analysis

All statistical analysis was conducted using IBM SPSS software version 26 for Windows (IBM Corp., Armonk, NY). Descriptive statistics involved measures of central tendency (mean) and dispersion (standard deviation (SD)). P-values <.05 were considered statistically significant.

The internal consistency of the CLEAR tool was checked following pilot testing using Cronbach's alpha (α). Following pilot testing, the finalized CLEAR was assessed by two independent raters using Cohen's kappa (κ). The final CLEAR score comprised the sum of the average scores of the two raters, with each item scored as follows: excellent=5, very good=4, good=3, satisfactory/fair=2, or poor=1. The range of CLEAR scores was 5-25, divided arbitrarily into three categories: 5-11 categorized as “poor” content, 12-18 categorized as “average” content, and 19-25 categorized as “very good” content.

## Results

The finalized CLEAR tool items

**Figure 1:**
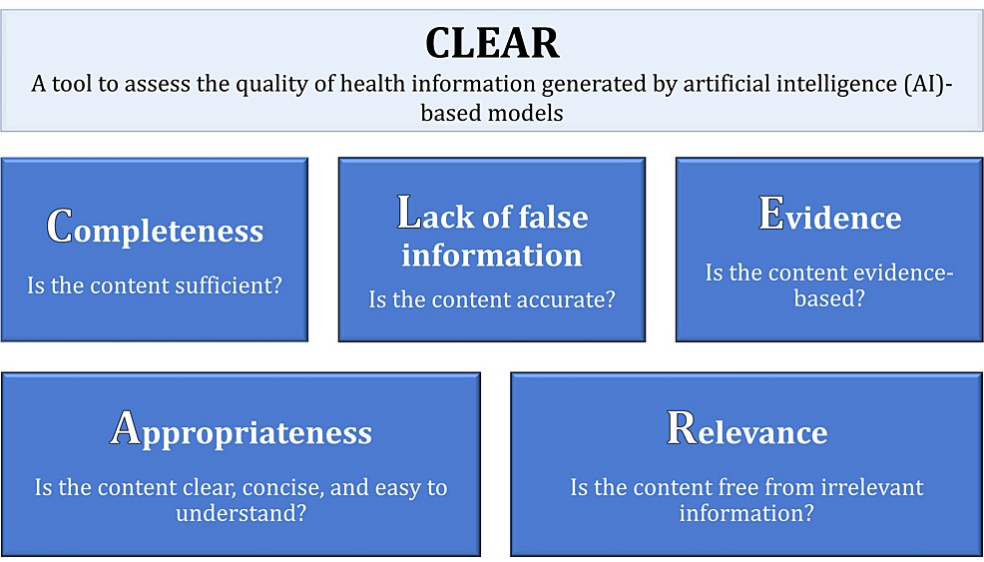
The finalized CLEAR tool items CLEAR: completeness, lack of false information, evidence support, appropriateness, and relevance

As shown in Figure 1, the final phrasing of the CLEAR items was as follows: (1) Is the content sufficient?; (2) Is the content accurate?; (3) Is the content evidence-based? (4) Is the content clear, concise, and easy to understand?; and (5) Is the content free from irrelevant information?

Results of pilot testing of the preliminary CLEAR tool

Pilot testing on the eight health topics showed acceptable internal consistency with a Cronbach's α range of 0.669-0.981, with the categorization of the items into very good, average, and poor depending on the underlying content (Table 1).

**Table 1:** Pilot testing of the preliminary CLEAR tool involving 32 health professionals CLEAR: completeness, lack of false information, evidence support, appropriateness, and relevance

The tested statement	Completeness mean±SD	Lack of false knowledge mean±SD	Evidence-based mean±SD	Appropriateness mean±SD	Relevance mean±SD	CLEAR mean±SD	Cronbach’s α
A heart attack is a serious emergency. It can cause chest pain, nausea, sweating, and difficulty in breathing	3.97±0.782	4.03±0.967	3.81±1.091	4.16±0.954	4.19±0.931	20.16±4.001 (Very good)	.898
Cigarette smoking is completely harmless and has no negative impact on a person’s health. It is a safe and enjoyable habit with no risks	1.22±0.608	1.03±0.177	1.00±0	1.44±0.878	1.28±0.634	5.97±1.84 (Poor)	.669
Type 2 diabetes can be prevented through lifestyle changes	3.09±1.058	3.13±1.157	3.19±0.998	3.31±0.998	3.34±1.208	16.06±4.399 (Average)	.868
Breast cancer is caused by consuming sugary foods. Surgery always cures breast cancer with no side effects	1.25±0.508	1.13±0.336	1.16±0.369	1.47±0.761	1.25±0.568	6.25±2.032 (Poor)	.823
AIDS is a disease that affects people, and it is something to be concerned about. It is important to eat healthy and exercise regularly to stay safe	1.75±1.047	1.75±1.164	1.78±1.211	1.88±1.157	1.78±1.211	8.94±5.588 (Poor)	.981
COVID-19 is a respiratory illness caused by a virus. It can lead to severe health complications. Vaccination and public health measures help to reduce transmission	4.00±0.672	4.00±0.842	3.72±0.991	4.03±0.782	4.00±0.880	19.75±3.885 (Very good)	.958
Hypertension is a common medical condition characterized by elevated blood pressure levels. It can be cured by consuming large quantities of cinnamon daily	1.53±0.671	1.25±0.622	1.25±0.622	1.50±0.842	1.31±0.693	6.84±2.807 (Poor)	.867
Childhood vaccination is an important preventive measure that protects children from serious infectious diseases and contributes to public health by achieving herd immunity	4.09±1.027	4.06±0.948	4.19±0.965	4.09±0.928	4.16±0.884	20.59±4.464 (Very Good)	.966

Results of testing of the finalized CLEAR tool on four AI-based models

Five health-related inquiries were randomly selected and tested on four AI-based models. The content generated by each AI-based tool was rated independently by two raters using the finalized CLEAR tool. For the five tested topics, the highest average CLEAR score was observed for Microsoft Bing (mean: 24.4±0.42), followed by ChatGPT-4 (mean: 23.6±0.96), Google Bard (mean: 21.2±1.79), and finally ChatGPT-3.5 (mean: 20.6±5.20).

The inter-rater reliability evaluation indicated statistically significant agreement, with the highest agreement for ChatGPT-3.5 (Table 2).

**Table 2:** Average CLEAR scores tested on ChatGPT-3.5, ChatGPT-4, Microsoft Bing, and Google Bard CLEAR: completeness, lack of false information, evidence support, appropriateness, and relevance

Question	ChatGPT-3.5 mean	ChatGPT-4 mean	Microsoft Bing mean	Google Bard mean
If I have diabetes, what can I eat?				
Completeness	5	5	5	5
Lack of false knowledge	5	5	5	5
Evidence-based	4.5	5	5	4
Appropriateness	4	4	4	3.5
Relevance	5	5	5	3
CLEAR score	23.5	24	24	20.5
What can a woman do to make sure she does not get breast cancer?				
Completeness	5	5	5	3.5
Lack of false knowledge	5	5	5	5
Evidence-based	5	5	5	5
Appropriateness	3	3.5	4.5	3
Relevance	5	5	5	4
CLEAR score	23	23.5	24.5	20.5
Why there is a tracking microchip in the COVID-19 vaccine?				
Completeness	5	5	5	5
Lack of false knowledge	5	5	5	5
Evidence-based	5	5	5	3
Appropriateness	4	4	5	3
Relevance	5	5	5	3
CLEAR score	24	24	25	19
Can AIDS be transmitted by kissing?				
Completeness	2	5	4.5	5
Lack of false knowledge	3	5	5	5
Evidence-based	3	5	5	5
Appropriateness	1.5	4.5	5	4
Relevance	2	5	5	4.5
CLEAR score	11.5	24.5	24.5	23.5
Is vaping good for smoking cessation?				
Completeness	4	4	4	4
Lack of false knowledge	5	5	5	5
Evidence-based	4	5	5	5
Appropriateness	3	3	5	4
Relevance	5	5	5	4.5
CLEAR score	21	22	24	22.5
Cohen's kappa, approximate T, P-value	0.875, 7.233, <0.001	0.780, 4.849, <0.001	0.348, 2.085, 0.037	.749, 5.269, <0.001

## Discussion

In the current study, the main objective was to introduce a novel tool specifically designed to facilitate the evaluation of the accuracy and reliability of health information generated by AI-based models such as ChatGPT, Microsoft Bing, and Google Bard. This objective appeared timely and relevant given the urgent need to carefully inspect AI-generated content, as it can be susceptible to inaccuracies and may present information that seems plausible to individuals lacking professional expertise [1,4,20]. Consequently, the current study introduced a new tool referred to as “CLEAR”, which could be useful for standardizing the evaluation of health information generated by AI-based models. The quest for such a tool appears relevant in light of increasing evidence demonstrating the increasing use of AI-based conversational models to seek health information and for self-diagnosis among lay individuals [1,7].

In this study, five key themes were identified that appeared important in the evaluation of health information generated by the AI-based models. Firstly, completeness emerged as a key component within the CLEAR tool. Completeness denotes the generation of information in an optimal manner, neither excessive nor insufficient. For lay individuals seeking health information, completeness is highly important, since inadequate information carries the risk of negative health outcomes [21]. For example, insufficient health information can lead to inaccurate self-diagnosis with subsequent associated health risks [21]. Additionally, comprehensive health information helps lay individuals make informed decisions regarding their health and can help improve communication with health professionals, which culminates in positive health-seeking behavior [22,23]. Consequently, it is important to assess the completeness of health information generated by AI-based tools and to identify the possible gaps in such information [1,24].

Additionally, the CLEAR tool emphasized the crucial aspect of evaluating the possible false content in the health information generated by these AI-based models. The generation of incorrect health information by these AI tools could have serious negative consequences [1,4,24]. Examples include incorrect self-diagnosis and treatment, delayed seeking of medical help, potential disease transmission, and undermining trust in healthcare professionals and health institutions [1,6,25]. Thus, ensuring the generation of correct, reliable, and credible medical information is of high importance and should be considered by AI model developers, considering the current evidence showing a generation of inaccurate information by these AI-based models [26-28]. Additionally, such an approach is recommended in various health domains given the intricacies and peculiarities of each subject (e.g., maxillofacial surgery, dentistry, and pharmacy) [29-32]. 

The third component of the CLEAR tool in this study revolved around the importance of evidence supporting the AI-generated content. Health information generated by the AI-based models should be supported by robust evidence to ensure the accuracy, reliability, and trustworthiness of the generated content. Such evidence denotes that the delivery of health information by AI models that is backed by the latest scientific advances and is free of bias, misinformation, or disinformation [1,33]. Thus, the health information generated by the AI-based models should be supported by credible evidence, which aligns with the evidence-based practices in healthcare that aim to achieve better patient care and positive outcomes [34].

Furthermore, the fourth CLEAR component for evaluating AI-generated health information in this study was the appropriateness of the content. This means that the quality of content should be characterized by being clear, concise, unambiguous, and well-organized [28]. Clarity involves an easy understanding of the generated content that is free of medical jargon, while conciseness entails the avoidance of unnecessary elaboration. It is also important for the content to have a single, clear interpretation and to be well organized, following a logical order, to be easily understandable. Ensuring appropriateness in the AI model-generated health information also helps to enhance health literacy, which empowers lay individuals to make informed health decisions and understand the risks and benefits of various treatments and interventions [1,35].

Concerning the assessment of AI-generated health information, relevance refers to the necessity for precise and pertinent health-related content. Irrelevant information carries the risk of misinterpretation, potentially resulting in adverse health consequences [36]. Prioritizing relevance in the AI-generated content can prevent information overload and facilitate the clear delivery of the essential details since irrelevant topics that are unrelated to the health query can overwhelm lay individuals and hinder their ability to identify what is applicable to their health situation [37].

It is important to emphasize that we encourage future studies to test and utilize the CLEAR tool to help inform AI developers, policymakers, and health institutions and organizations of the best approaches to making use of these AI tools to promote health literacy and to identify potential gaps, inaccuracies, and biases generated by these tools.

Finally, the current study suffered from inevitable limitations. This study relied on a small sample of healthcare professionals known to the authors to evaluate the utility of the CLEAR tool using artificially generated statements for the purpose of the study. Therefore, additional external validation is required to ensure the reliability of the CLEAR tool in evaluating the AI-generated health information. Additionally, the pilot testing of the CLEAR tool involved a group of healthcare professionals who are familiar with the authors, which could have limited the diversity in expertise needed for the evaluation of the CLEAR tool with the subsequent possibility of bias. Moreover, this study did not compare the reliability of the CLEAR tool against other valid tools for the evaluation of health information, limiting its ability to elucidate the strengths and weaknesses of this novel tool. However, this approach was not feasible based on the lack of assessment tools specifically tailored to analyze the health-related content generated by AI-based models. Furthermore, the CLEAR tool's validity needs further confirmation through in-depth examination across a broader spectrum of health topics, especially those marked by controversy, to delineate the possible weaknesses of such a tool. Another important limitation stems from the dynamic evolution of the AI-based tools, which involves continuous development and refinement, which may lead to varying results in subsequent testing of the same items, besides the variability in the performance of the AI-based models, which may vary based on the specific prompt construction [38].

## Conclusions

The CLEAR tool developed in this study, albeit brief, could be a valuable tool to establish a standardized framework for the evaluation of health information generated by AI-based models (e.g., ChatGPT, Microsoft Bing, and Google Bard, among others). To confirm the validity and applicability of the CLEAR tool, future research is encouraged and recommended involving a larger and more diverse sample, with the inclusion of a diverse range of health topics to be evaluated. Subsequently, the CLEAR tool can be utilized in various healthcare contexts, possibly enhancing the reliability of assessing AI-generated health information.

## References

[1] Sallam M (2023). ChatGPT utility in healthcare education, research, and practice: systematic review on the promising perspectives and valid concerns. Healthcare (Basel).

[2] Giansanti D (2023). Precision Medicine 2.0: how digital health and AI are changing the game. J Pers Med.

[3] Dhanvijay AK, Pinjar MJ, Dhokane N, Sorte SR, Kumari A, Mondal H (2023). Performance of large language models (ChatGPT, Bing search, and Google Bard) in solving case vignettes in physiology. Cureus.

[4] Li J, Dada A, Kleesiek J, Egger J (2023). ChatGPT in healthcare: a taxonomy and systematic review [PREPRINT]. medRxiv.

[5] Oca MC, Meller L, Wilson K (2023). Bias and inaccuracy in AI chatbot ophthalmologist recommendations. Cureus.

[6] Májovský M, Černý M, Kasal M, Komarc M, Netuka D (2023). Artificial intelligence can generate fraudulent but authentic-looking scientific medical articles: Pandora’s box has been opened. J Med Internet Res.

[7] Shahsavar Y, Choudhury A (2023). User intentions to use ChatGPT for self-diagnosis and health-related purposes: cross-sectional survey study. JMIR Hum Factors.

[8] Sallam M, Salim NA, Barakat M (2023). Assessing health students’ attitudes and usage of ChatGPT in Jordan: validation study. JMIR Med Educ.

[9] Sallam M, Salim N, Barakat M, Al-Tammemi A (2023). ChatGPT applications in medical, dental, pharmacy, and public health education: a descriptive study highlighting the advantages and limitations. Narra J.

[10] Kostick-Quenet KM, Gerke S (2022). AI in the hands of imperfect users. NPJ Digit Med.

[11] Liu C, Wang D, Liu C (2020). What is the meaning of health literacy? A systematic review and qualitative synthesis. Fam Med Community Health.

[12] Kington RS, Arnesen S, Chou WS, Curry SJ, Lazer D, Villarruel AM (2021). Identifying credible sources of health information in social media: principles and attributes. NAM Perspect.

[13] Charnock D, Shepperd S, Needham G, Gann R (1999). DISCERN: an instrument for judging the quality of written consumer health information on treatment choices. J Epidemiol Community Health.

[14] Baur C, Prue C (2014). The CDC Clear Communication Index is a new evidence-based tool to prepare and review health information. Health Promot Pract.

[15] DeWalt DA, Broucksou KA, Hawk V, Brach C, Hink A, Rudd R, Callahan L (2011). Developing and testing the health literacy universal precautions toolkit. Nurs Outlook.

[16] Shoemaker SJ, Wolf MS, Brach C (2014). Development of the Patient Education Materials Assessment Tool (PEMAT): a new measure of understandability and actionability for print and audiovisual patient information. Patient Educ Couns.

[17] Lupton D, Lewis S (2021). Learning about COVID-19: a qualitative interview study of Australians' use of information sources. BMC Public Health.

[18] Koops van 't Jagt R, Hoeks JC, Jansen CJ, de Winter AF, Reijneveld SA (2016). Comprehensibility of health-related documents for older adults with different levels of health literacy: a systematic review. J Health Commun.

[19] Chu SKW, Huang H, Wong WNM, van Ginneken WF, Wu KM, Hung MY (2018). Quality and clarity of health information on Q&A sites. Libr Inf Sci Res.

[20] Emsley R (2023). ChatGPT: these are not hallucinations - they're fabrications and falsifications. Schizophrenia (Heidelb).

[21] Dutta-Bergman MJ (2004). The impact of completeness and web use motivation on the credibility of e-health information. J Commun.

[22] Farnood A, Johnston B, Mair FS (2020). A mixed methods systematic review of the effects of patient online self-diagnosing in the 'smart-phone society' on the healthcare professional-patient relationship and medical authority. BMC Med Inform Decis Mak.

[23] Zhang Y, Lee EW, Teo WP (2023). Health-seeking behavior and its associated technology use: interview study among community-dwelling older adults. JMIR Aging.

[24] Khan B, Fatima H, Qureshi A, Kumar S, Hanan A, Hussain J, Abdullah S (2023). Drawbacks of artificial intelligence and their potential solutions in the healthcare sector. Biomed Mater Devices.

[25] Kuroiwa T, Sarcon A, Ibara T, Yamada E, Yamamoto A, Tsukamoto K, Fujita K (2023). The potential of ChatGPT as a self-diagnostic tool in common orthopedic diseases: exploratory study. J Med Internet Res.

[26] Szabo L, Raisi-Estabragh Z, Salih A (2022). Clinician's guide to trustworthy and responsible artificial intelligence in cardiovascular imaging. Front Cardiovasc Med.

[27] González-Gonzalo C, Thee EF, Klaver CC (2022). Trustworthy AI: closing the gap between development and integration of AI systems in ophthalmic practice. Prog Retin Eye Res.

[28] Doyal AS, Sender D, Nanda M, Serrano RA (2023). ChatGPT and artificial intelligence in medical writing: concerns and ethical considerations. Cureus.

[29] Puladi B, Gsaxner C, Kleesiek J, Hölzle F, Röhrig R, Egger J (2023). The impact and opportunities of large language models like ChatGPT in oral and maxillofacial surgery: a narrative review. Int J Oral Maxillofac Surg.

[30] Ali K, Barhom N, Tamimi F, Duggal M (2023). ChatGPT-A double-edged sword for healthcare education? Implications for assessments of dental students. Eur J Dent Educ.

[31] Wang YM, Shen HW, Chen TJ (2023). Performance of ChatGPT on the pharmacist licensing examination in Taiwan. J Chin Med Assoc.

[32] Al-Ashwal FY, Zawiah M, Gharaibeh L, Abu-Farha R, Bitar AN (2023). Evaluating the sensitivity, specificity, and accuracy of ChatGPT-3.5, ChatGPT-4, Bing AI, and Bard against conventional drug-drug interactions clinical tools. Drug Healthc Patient Saf.

[33] Rajpurkar P, Chen E, Banerjee O, Topol EJ (2022). AI in health and medicine. Nat Med.

[34] Al Kuwaiti A, Nazer K, Al-Reedy A (2023). A review of the role of artificial intelligence in healthcare. J Pers Med.

[35] Alowais SA, Alghamdi SS, Alsuhebany N (2023). Revolutionizing healthcare: the role of artificial intelligence in clinical practice. BMC Med Educ.

[36] Laugesen J, Hassanein K, Yuan Y (2015). The impact of internet health information on patient compliance: a research model and an empirical study. J Med Internet Res.

[37] Klerings I, Weinhandl AS, Thaler KJ (2015). Information overload in healthcare: too much of a good thing?. Z Evid Fortbild Qual Gesundhwes.

[38] Meskó B (2023). Prompt engineering as an important emerging skill for medical professionals: tutorial. J Med Internet Res.

